# Multiplex flow cytometry-based assay for quantifying tumor- and virus-associated antibodies induced by immunotherapies

**DOI:** 10.3389/fimmu.2022.1038340

**Published:** 2022-11-16

**Authors:** Jessica A. Minott, Jacob P. van Vloten, Jacob G. E. Yates, Lily Chan, Geoffrey A. Wood, Alicia M. Viloria-Petit, Khalil Karimi, James J. Petrik, Sarah K. Wootton, Byram W. Bridle

**Affiliations:** ^1^ Department of Pathobiology, University of Guelph, Guelph, ON, Canada; ^2^ Department of Biomedical Sciences, University of Guelph, Guelph, ON, Canada; ^3^ ImmunoCeutica Inc., Cambridge, ON, Canada

**Keywords:** antibody response, immunotherapy, flow cytometry, oncolytic virus, virus-vectored vaccines

## Abstract

Novel immunotherapies continue to be developed and tested for application against a plethora of diseases. The clinical translation of immunotherapies requires an understanding of their mechanisms. The contributions of antibodies in driving long-term responses following immunotherapies continue to be revealed given their diverse effector functions. Developing an in-depth understanding of the role of antibodies in treatment efficacy is required to optimize immunotherapies and improve the chance of successfully translating them into the clinic. However, analyses of antibody responses can be challenging in the context of antigen-agnostic immunotherapies, particularly in the context of cancers that lack pre-defined target antigens. As such, robust methods are needed to evaluate the capacity of a given immunotherapy to induce beneficial antibody responses, and to identify any therapy-limiting antibodies. We previously developed a comprehensive method for detecting antibody responses induced by antigen-agnostic immunotherapies for application in pre-clinical models of vaccinology and cancer therapy. Here, we extend this method to a high-throughput, flow cytometry-based assay able to identify and quantify isotype-specific virus- and tumor-associated antibody responses induced by immunotherapies using small sample volumes with rapid speed and high sensitivity. This method provides a valuable and flexible protocol for investigating antibody responses induced by immunotherapies, which researchers can use to expand their analyses and optimize their own treatment regimens.

## Introduction

Knowledge of the immune system’s intrinsic ability to recognize and destroy infectious pathogens and malignant cells has paved the way for attempts to control numerous diseases by immunological means, thereby advancing the field of immunotherapy to what it is today. Many immunotherapies are predicated on exploiting the specificity and selectivity of host immune responses to fight disease, and function by improving the quality and/or quantity of immunological effector mechanisms against a desired target to reduce the severity of clinical disease. The armamentarium that broadly fulfills the definition of immunotherapeutic agents is extensive, including but not limited to, cancer vaccines, virus-vectored vaccines, oncolytic viruses (OVs), monoclonal antibodies, immune checkpoint inhibitors and more, with each uniquely positioned to enhance the built-in protective mechanism of host immune responses against several public health concerns (1–8).

The means by which the immune system identifies and eliminates pathogens or neoplastic cells are complex, and include the involvement of cellular and humoral effectors to drive protective responses (9–11). Historically, many immunotherapies, particularly against cancers, have focused on eliciting and evaluating cytotoxic T cell responses (12–14). Many studies however, particularly in the field of infectious diseases, continue to reveal how important the contributions of other immunological effector molecules are in driving therapeutically beneficial responses. Among the various molecules involved in mediating long-term protection to disease are antibodies, which possess broad effector functions. Target cell death can be induced through the direct binding of antibodies to surface-expressed antigens, which can result in obstruction of crucial downstream signaling cascades (15), or neutralization of infectivity in the case of viral infections (16, 17). Antibodies can also mediate target cell death indirectly through Fc receptor-mediated phagocytosis, antibody-dependent cellular cytotoxicity, or complement-mediated lysis (18–20). Subclasses of immunoglobulin isotypes possess distinct immunological functions, and exhibit discrete clinical effects following immunotherapy, particularly in the context of cancers (21–23). For example, IgG4 antibodies have been found to impair IgG1-mediated antitumor immunity, promote T-helper cell-2-biased inflammation, and shorten survival times for patients with melanomas, breast, pancreatic, or gastric cancers (24–26).

Given the potential involvement of antibody responses in mediating disease outcomes, it is important to identify therapy-induced subclasses of immunoglobulins (Igs) that contribute towards a tailored and maximally protective effect following immunotherapy. As such, methods that measure antibody responses to various classes of immunotherapies may prove key to improving efficacy. We previously developed a flexible methodology for detecting antibody responses generated by antigen-agnostic immunotherapies (27). Here, we expand this method to a high-throughput, multiplex, flow cytometry assay positioned to resolve current challenges in antibody detection, such as high selectivity and sensitivity, low operation cost, limited sample requirement and simple and rapid detection procedures. Our protocol makes use of antigen-expressing target cells as reservoirs to bind multiple isotypes of sample-derived antibodies associated with a given immunotherapeutic platform. These antibodies are subsequently detected and quantified using fluorochrome-conjugated detection antibodies and standardized beads.

The protocol herein provides an advantageous method for assessing broad endogenous antibody responses against multiple antigenic targets following immunotherapy. By facilitating detection of a variety of isotypes of therapy-induced antibodies, the presented methodology can be used to dissect primary antibody responses, which are often of low magnitude to tolerized tumor antigens following many cancer immunotherapies, and subsequent secondary antibody responses in patients that may have pre-existing antibodies at the time of initial treatment or that receive multiple rounds of treatment. Additionally, this protocol can be extended to analyze immunoglobulin class switching and type 1 versus type 2 immune response biases elicited by a given immunotherapy platform throughout the course of the antibody response. In turn, this can improve the current understanding of both the quantitative and qualitative nature of immunotherapy-induced antibody responses.

## Materials and methods

### Reagents list

#### Retro-orbital blood draw:

o Heparinized microhematocrit capillary tubes to collect plasma (Fisher Scientific, MA, USA, catalog number [Cat#] 22-362-566). Alternatively, serum from clotted blood can be used, but this would restrict analyses to serum-derived factors only. Use of unclotted blood facilitates simultaneous cellular analyses.
o 1.5 mL microfuge tubes
o Gauze pads
o Eye Lubricant
o Container filled with ice pellets

#### Peritoneal lavage:

o Gauze pads
o 3 mL syringes
o BD PrecisionGlide™ Single-use Needles (Fisher Scientific, Cat#14-821-13G)
o 1.5 mL microfuge tubes
o Phosphate-buffered saline (PBS; Fisher Scientific, Cat#SH30256.01)
o Container filled with ice pellets

#### Cell culture:

o DF-1 (immortalized chicken embryonic fibroblasts; American Type Culture Collection [ATCC] CRL-12203), Vero (African green monkey kidney; ATCC CCL-81) and ID8 (murine ovarian carcinoma; generously donated by Drs. K. Roby and P. Terranova, Kansas State University, Manhattan, KS) cells
o Complete Dulbecco’s modified eagle’s medium (DMEM) (Fisher Scientific, Cat#SH30022.01) or media specific to target cell line of interest
  o 10% fetal bovine serum (VWR, PA, USA, Cat#97068-085)
  o Penicillin/Streptomycin cocktail (Fisher Scientific, Cat#SV30010)
  o 1x non-essential amino acids (Fisher Scientific, Cat#11140050)
o 0.25% Ethylene-diamine-tetra-acetic acid (EDTA; Corning, NY, USA, Cat#25-052-CI)
o PBS
o Gibco™ Trypan Blue Solution, 0.4% (Fisher Scientific, Cat#15250061)
o MycoAlert PLUS Mycoplasma Detection Kit (Lonza, Basel Switzerland, Cat# LT07-703)
o Cell culture-treated flasks and 96-well U-bottom plates (Fisher Scientific, Cat# 12-565-65). 1x10^5^ target cells per well of a 96-well U bottom plate is optimal for antibody analysis. The total number of cells required will depend on the chosen sample dilution series and the total number of samples. Total target cell numbers needed should be determined ahead of time to prepare the appropriate number of T75cm^2^ flasks to facilitate target cell growth.
o 50 mL conical tubes (Fisher Scientific, Cat# 14-432-22)
o Multi-channel and standard 10-50 µL and 30-300 µL pipettes

#### Flow cytometry:

o Fluorescence-activated cell sorting (FACS) tubes (Falcon round- bottom polystyrene tubes, Corning, Cat# 14-959-5)
o Bovine serum albumin (BSA; Fisher Scientific, Cat#BP1600100)
o FACS buffer (PBS + 0.5% BSA)
o Hank’s Balanced Salt Solution (HBSS; Fisher Scientific, Cat#SH3003103)
o Fixation buffer (BioLegend, CA, USA, Cat#420801)
o Intracellular staining permeabilization wash buffer (BioLegend, Cat#421002)
o Quantum Molecule of Equivalent Soluble Fluorophore (MESF) Bead Kit (Bang Laboratories, Cat#488). The choice of kit will vary depending on the fluorochromes of the secondary antibody conjugates being utilized. Manufacturer recommendations should be followed to set up for analysis.
o Murine Antibodies
  - IgG1 - Alexa Fluor 488 (BioLegend, Cat#406625)
  - IgG1 - Allophycocyanin (APC) (BioLegend, Cat#406610)
  - IgG2a/c - Alexa Fluor 488 (BioLegend, Cat#407122)
  - IgG2b - Phycoerythrin cyanine 7 (PE-Cy7) (BioLegend, Cat#406713)
  - IgM - Peridinin chlorophyll protein complex (PerCP-Cy5.5) (BioLegend, Cat#406511)
  - IgA – Brilliant violet (BV) 421 (BD Biosciences, Cat#743293)

### Equipment list

o Biological safety cabinet – for all steps which involve sterile cell culture and sample processing
o Humidified incubator (5% CO_2_ and 37.0°C)
o Standard centrifuges
o Anesthetic machine
o Microscope capable of brightfield and fluorescence
o A hemocytometer counting chamber device
o A flow cytometer capable of detecting up to eight colors is optimal. A three-laser, eight-color FACS Canto II with FACSDiva software (BD Biosciences, Ontario, Canada) was used to generate the data shown here. Manufacturer recommendations should be followed to set up the flow cytometer for multi-color analysis.
o FlowJo (BD BioSciences, Ontario, Canada) and GraphPad Prism (GraphPad Software
San Diego, California) software was used to analyze and graph, respectively, the flow cytometry data presented here.

### Stepwise procedure

**Figure 1:**
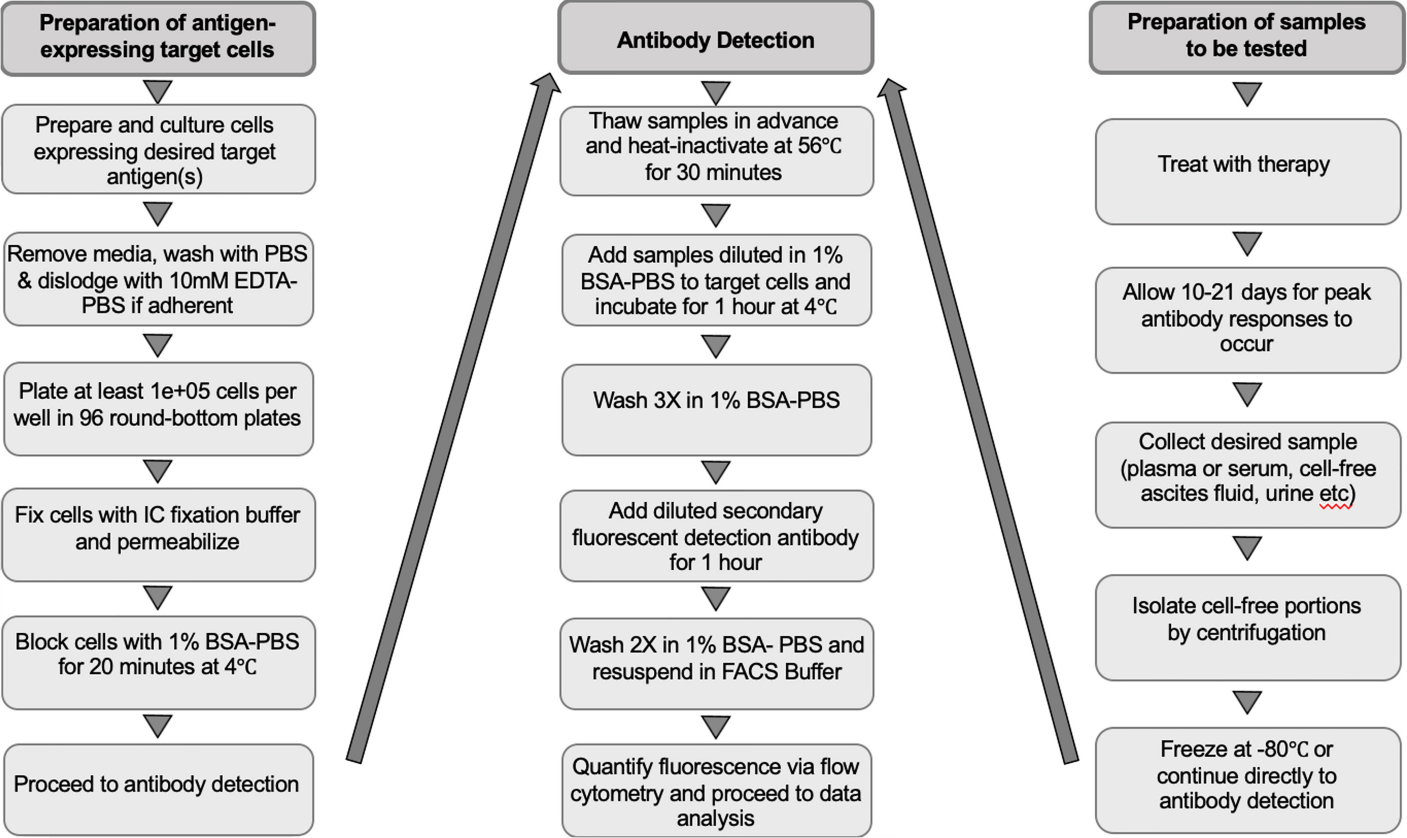
Workflow of the experimental procedure for preparing target cells, collecting samples to be tested and detecting immunotherapy-induced antibodies. EDTA-PBS, ethylenediaminetetra-acetic acid-phosphate-buffered saline; BSA-PBS, bovine serum albumin-phosphate buffered saline; FACS, fluorescence-activated cell scanning; IC, intracellular.

o For an experimental workflow, refer to **Figure 1**.

#### Preparation of target cells

**Figure 2:**
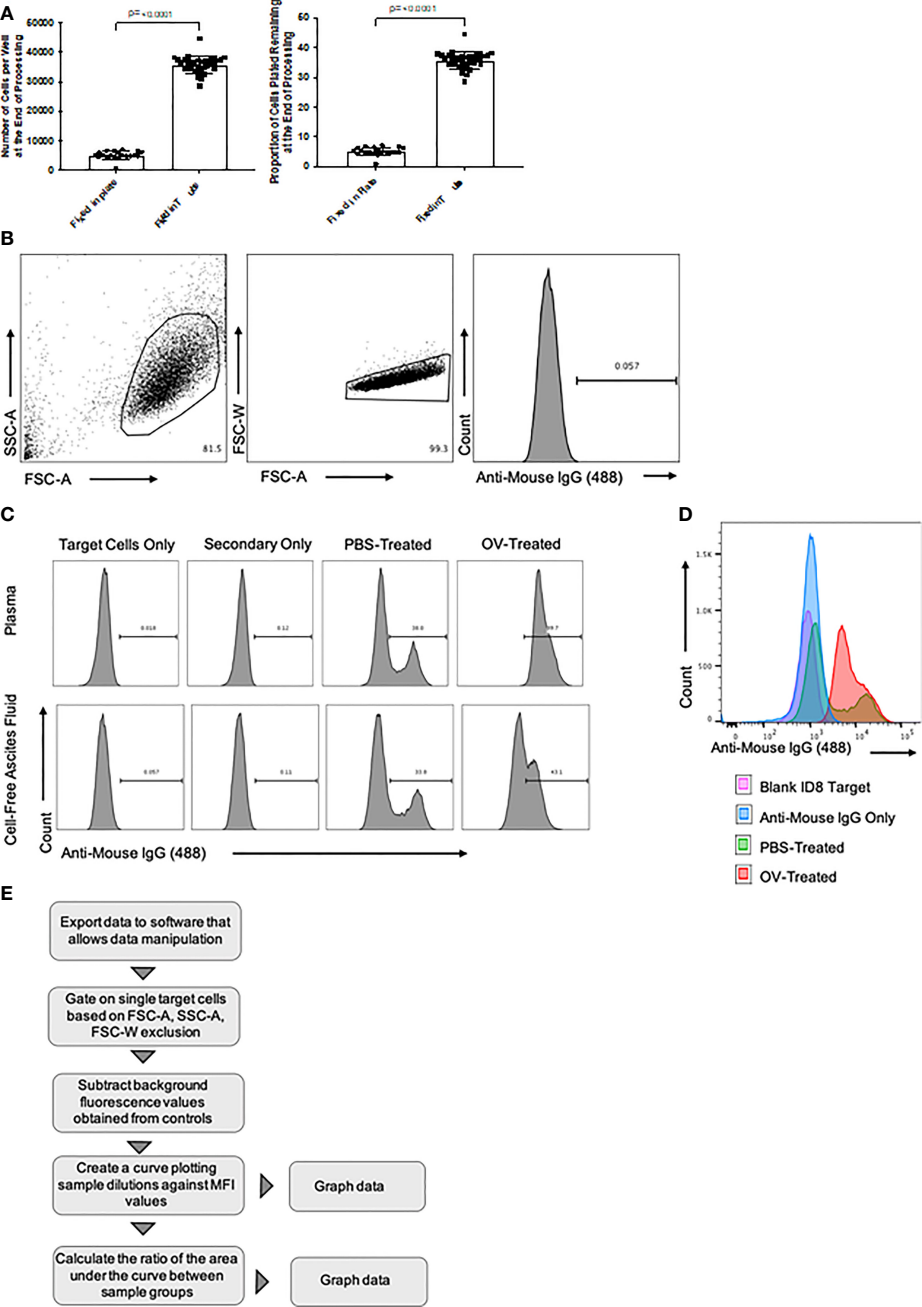
Overview of flow cytometry optimization, gating strategy, and data analysis. **(A)** Target cell recovery following fixation of cells in fluorescence-activated cell sorting (FACS) tubes versus 96-well U-bottom plates. The number of cells recovered, and the proportion of cells remaining following processing, at the time of target-directed antibody detection are shown. Statistical analysis was conducted using a two-tailed Student’s t-test. Statistical significance was defined as P-values less than or equal to 0.05. **(B)** Targeted ID8 tumor cells were gated based on forward scatter area (FSC-A) versus side scatter area (SSC-A) characteristics (left panel). Doublets were then removed after gating FSC-A versus forward scatter width (FSC-W) (middle panel). Histograms were generated by plotting the cell count on the y-axis versus anti-mouse IgG1-Alexa Fluor 488 secondary antibody-mediated fluorescence on the x-axis (right panel). **(C)** Generated histograms representative of tumor-associated antibodies binding to target ID8 murine ovarian carcinoma cells in either plasma (top panels) or cell-free ascites fluid (bottom panels) samples diluted 1/100. These samples were collected from ID8 tumor-bearing mice 21 days after treatment with either phosphate-buffered saline (PBS) or an oncolytic virus (OV). Histograms generated from intra-assay target cell and secondary antibody only controls identified background fluorescence. Tumor-associated antibody binding was represented as a function of positive anti-mouse IgG1 Alexa Fluor 488 conjugated secondary antibody signals from target ID8 tumor cells incubated with plasma from either PBS or OV-treated ID8 tumor-bearing mice. **(D)** Representative histogram overlay comparing an anti-mouse IgG1 Alexa Fluor 488 signal from targeted ID8 tumor cells treated with 1% BSA alone (pink), anti-mouse IgG Alexa Fluor 488 alone (blue), or cell-free ascites fluid diluted 1/100 from PBS-treated (green) or OV-treated (red) ID8 tumor-bearing mice and then stained with anti-mouse IgG1 Alexa Fluor 488. **(E)** Data analysis workflow summary, from data export to graphical representation. MFI, mean fluorescence intensity.

- Culture target cells in flasks or plates to 80%-90% confluency
  o Tissue-culture treated flasks and plates were used for adherent cell lines.
  o Cell lines for this research were either gifted or obtained directly from ATCC. To assure reproducibility, cell lines were expanded in isolation from other cell lines immediately upon arrival, and many aliquots were frozen to create a low-passage lab stock from which project-specific stocks were made. All cell lines were confirmed to be mycoplasma-free using a MycoAlert PLUS Mycoplasma Detection Kit.
  o When using this assay to assess tumor-associated antibody responses, using autologous tumor cells as the target cell type allows for every relevant tumor antigen not generated *de novo* to be represented. This facilitates detection of the full breadth of the antibody response induced by the immunotherapy.
  o For determining non-tumor antigen-specific therapy-induced antibodies, non-tumor permissive cells expressing the relevant target antigen(s) were obtained by infection with an antigen-expressing viral platform distinct from the viral-vectored vaccine platform used for treatment. Where applicable, a standard immunofluorescence assay (IFA) can be performed to determine if there is a sufficient concentration of the target antigen expressed on cells.
- Remove culture medium and wash cells in a large volume of PBS (*e.g.*, 10 mL per T75cm^2^ flask)
- If adherent, detach cells using 10 mM EDTA-PBS, and incubate for 10 minutes at 37°C
  o *EDTA-PBS is used in replacement of trypsin to dislodge adherent cells while preserving the expression of trypsin-sensitive antigens on the cell surface*.
- Resuspend cells in culture medium and enumerate using a counting chamber.
- Centrifuge cells at 500x*g* for five minutes and discard supernatant.
- Resuspend cells in 50 μL of fixation buffer per 1x10^5^ cells and incubate for 15 minutes at room temperature.
  o Target cell recovery was observed to be optimal when cells were fixed in a tube prior to plating (**Figure 2A**). Rock the tube gently on a shaker during the fixation step to prevent cells from fixing to the sides of the tube.
- Add 150 μL of permeabilization buffer per 1x10^5^ cells. Resuspend cells by pipetting well.
- Centrifuge at 500x*g* for five minutes, discard supernatant and resuspend cells in a volume of permeabilization buffer that will allow 1x10^5^ cells to be plated in 100 μL per well.
- Seed 1x10^5^ target cells per well in a 96-well U-bottom tissue culture plate
- *Pause Point:* Because the cells have been fixed, plates may be stored at 4°C while preparing sample dilutions
  o *We have stored plates for up to 24 hours without impacting the results*.

#### Sample collection and processing

o *Therapy- or tumor-directed antibody responses in these studies were quantified from blood or peritoneal lavages from tumor-free untreated or vaccinated mice, and PBS- or OV-treated ID8 tumor-bearing mice*

- Collect blood (100–200 μL is recommended if utilizing mice) and/or peritoneal lavage fluid in 1.5 mL microfuge tubes containing 5 μL of heparin (3 μg/mL diluted in HBSS) to prevent clotting and put on ice
  o It is recommended that the volume of sample harvested be enough to allow for preparation of all sample dilutions required, based on the maximum volume of sample allowed by the institutional animal care committee guidelines.
  o *The work presented here was approved by the University of Guelph Animal Care Committee (Animal Utilization Protocol #4662) and adhered to the policies published by the Canadian Council on Animal Care.*
- Transfer blood or peritoneal lavage fluid to FACS tubes and record the volume for each sample to facilitate normalizing data across samples on a per μL or per mL basis.
- Centrifuge at 500x*g* for 10 min at 4°C
  o Centrifugation of samples will separate plasma, or cell-free ascites fluid to the top layer. Collect clarified top layer without disturbing the cell pellet and aliquot into new 1.5 mL tubes.
- Heat-inactivate samples at 56°C for 30 min and then store on ice if continuing to step 3 or store the samples in an ultra-low temperature freezer, if using the pause point.
  o *This step enhances the sensitivity of antibody detection by eliminating complement proteins that could interfere with target antigens.*
- *Pause point:* Samples can be stored long-term at -80°C for future detection of antibody responses utilizing this procedure. Samples should be thawed on ice or at 4°C overnight prior to preparing sample dilutions.
  o *If analyzing antibody responses directly following sample collection and heat-inactivation, samples should be kept on ice before and during preparation of sample dilutions.*

#### Incubating target cells and antibody-containing samples

- Centrifuge plates containing fixed and permeabilized antigen-expressing target cells at 500x*g* for five minutes and discard supernatant by rapid inversion of the plate followed by blotting on absorbent paper, and then re-suspend the cells by gently tapping the side of the upright plate.
- Wash cells in 50 μL of PBS per well, repeat centrifugation and discard supernatant
- Block cells in 100 μL of 1% BSA-PBS per well and incubate for 20 min at 4°C
- *Pause point:* blocking can be done overnight at 4°Co
- Prepare dilutions of samples in 1% BSA-PBS in a 96-well plate for easy transfer
  o *A range of dilutions should be tested for each experiment to identify one that is optimal (the range will vary depending on the concentration of antibodies induced by a given therapy, with therapies capable of inducing more potent humoral responses requiring a greater dilution range). A six-dilution series was chosen in the experiments herein to generate accurate mean areas under the curve.*
- Remove the blocking solution by centrifuging plates containing target cells at 500x*g* for five minutes and discard supernatant
- Add diluted samples in 100 μL to target cell wells and incubate for one hour at 4°C
  o *Pause point*: Antibody-containing samples can be incubated with target cells overnight at 4°C
- Dilute wells with 100 μL of 1% BSA-PBS
- Centrifuge at 500x*g* for five minutes and discard supernatant
- Wash with 200 μL of 1% BSA-PBS
- Repeat the centrifugation and wash step
- Add all secondary detection antibodies at the recommended manufacturer or optimized dilutions in 1% BSA-PBS for a total of 100 μL per well.
  o *Antibodies should be titrated and tested at a range of dilutions to identify one that is optimal for positive signal detection.*
- Incubate for one hour at 4°C in the dark
- Dilute with 100 μL of 1% BSA-PBS
- Centrifuge at 500x*g* for five minutes and discard supernatant
- Wash with 200 μL of 1% BSA-PBS and repeat centrifugation and discard of supernatant
- Resuspend in 200 μL of FACS buffer for analysis on a flow cytometer

#### Quantum bead preparation

- *The procedure for preparing quantum beads was provided by Bang Laboratories, for use with the Quantum MESF Bead Kit (Cat#488). We advise optimizing the use of the desired microsphere standards in any application based on the manufacturer’s recommended conditions and protocols*.

#### Flow cytometry gating and data analysis

- Refer to **Figure 2**
- Data were analyzed using FlowJo software, but equivalent programs could be used. Target cells were gated based on forward scatter-area and side scatter-area characteristics. Doublets were then excluded by plotting forward scatter-area versus forward scatter-width. The presence of sample-derived antibodies bound to target cells were determined based on fluorescence emitted by secondary detection antibodies (**Figure 2B**). Histograms to assess the presence of immunotherapy-induced antibodies binding to target cells were generated by plotting the number of cells on the y-axis versus secondary detection antibody-mediated fluorescence on the x-axis (**Figure 2C**). Differences in fluorescence intensity between samples derived from control mice versus those that received an immunotherapy could be visualized by using the histogram overlay feature in the FlowJo software, as depicted in **Figure 2D**.
- Data were plotted on an x-y graph as mean fluorescence intensity (MFI) derived from a secondary detection antibody versus the number of target cells. This was done for all six dilutions of the tested sample. These data could then be plotted and used to calculate the area under the curve. The fold-change in area under the curve of treated samples compared to untreated samples could then be determined, following subtraction of the area under the curve for samples used to assess background fluorescence. A data analysis summary from data export to graphical representation of the results is provided in **Figure 2E**.

### Timing

*To maximize the sensitivity of this assay, samples of interest should be collected at the peak of the antibody response. Kinetics of peak antibody responses can be determined using this method by sampling on multiple days post-treatment. Following a kinetic analysis of the immune response, we chose day 21 post-treatment to quantify antibodies induced by our particular immunotherapy.*

Approximate time based on an experiment with 20 mice:

- Transduction/Plating of target cells: 24 hours
- Cell fixation and permeabilization: 30 minutes
- Blood or lavage fluid sampling: one hour
- Preparing sample dilutions: one hour
- Blocking and binding of antibody-containing samples: two hours (or overnight)
- Detection with secondary antibody: one-and-a-half hours
- Running samples on a flow cytometer: two hours

Total time from sample collection to data analysis: ~32 hours.

### Troubleshooting

#### Preparation of target cells

The outcome of this assay depends on the use of sufficient numbers of cells with preserved surface antigens that serve as reservoirs of the desired target antigens and can be detected on the flow cytometer. It is important to evaluate each target cell line for the optimal initial seeding density, growth kinetics and antigen expression (where applicable) to achieve adequate conditions at the time of the assay. The use of EDTA-PBS instead of trypsin-EDTA to detach adherent cells from plates allows for the preservation of trypsin-sensitive antigens on the surface of target cells. Together, these measures ensure a consistent number of available antigenic targets for all samples and minimizes background fluorescence otherwise caused by non-specific binding of secondary antibodies to the plate.

Incomplete monolayers or loss of cells during the experimental procedure can result in variability and artificially low on-target signals due to a reduction in the quantity of target antigens. To maximize adhesion and recovery of target cells to ensure high quality signals, cells were fixed in non-polymer-coated FACS tubes prior to plating to prevent fixing of cells to the sides of polymer-coated 96-well U-bottom plates (**Figure 2A**). Wells can be visualized by brightfield microscopy prior to flow cytometry to confirm adequate cell density, and any that do not meet quality control criteria should be excluded.

#### Preparation of internal experimental controls

##### Blank and ‘secondary antibody only’ background controls

Controls must be included in each experiment to support interpretation of results. It is relevant to include ‘target cell only’ controls, which do not include a sample or secondary antibodies, to facilitate appropriate gating during analysis of flow cytometry data, and to remove any background auto-fluorescence that may occur from the chosen cell line. Additionally, when quantifying immunotherapy-induced antibodies, target cells with only secondary antibodies are required to remove any non-antigen-specific background binding from all experimental data (**Figures 2C–E**)

##### Off-target cell controls

To identify potential antibody responses against antigens that are not target-specific, off-target controls that do not express the target antigen(s) can be utilized. These controls are important to include in each experiment to prove that antibody responses are truly therapy-induced and/or antigen or tumor-specific. For example, in the case of utilizing this assay to detect virus-associated antibodies following treatment with a virus-vectored vaccine platform, each experimental sample should be incubated with both virus-infected and uninfected cell controls. In the case of utilizing this assay to detect tumor-associated antibody responses, tumor cells that are different from those used for tumor implantation could be used to identify responses to antigens shared between cancer cells. Normal cell controls can also be used to detect antibodies that exist against non-cancer-specific antigens that may cross-react with target tumor cells. Therefore, it is important to include a sham-treated control group in each experiment to prove that the detected antibody responses are truly therapy-induced. During data analysis, positive secondary signals from the off-target cell controls would be categorized as ‘off-target background’ and should be subtracted from fluorescence values acquired from test wells.

##### Dilution of samples and secondary detection antibodies

The desired sample under investigation acts as the potential source of antigen-specific antibodies which bind to antigens in or on permeabilized target cells. Antibodies bound to antigens that are retained after washing are detected by isotype-specific secondary antibodies conjugated to fluorochromes. Induction of relatively low concentrations of antigen-specific antibodies is expected from many immunotherapies, particularly cancer-targeting platforms as they attempt to reactivate the immune system against self-derived, weakly immunogenic cancer antigens. This can be particularly problematic in scenarios where oncolytic viruses are the chosen therapeutic modality, given that more robust neutralizing antibody responses are frequently seen against the virus backbone-derived antigens. Thus, acquiring maximal volumes of blood from experimental animals will maximize the chance of detecting tumor-directed antibodies while facilitating splitting of samples for simultaneous assessment of antibodies induced against the viral vector.

**Figure 3:**
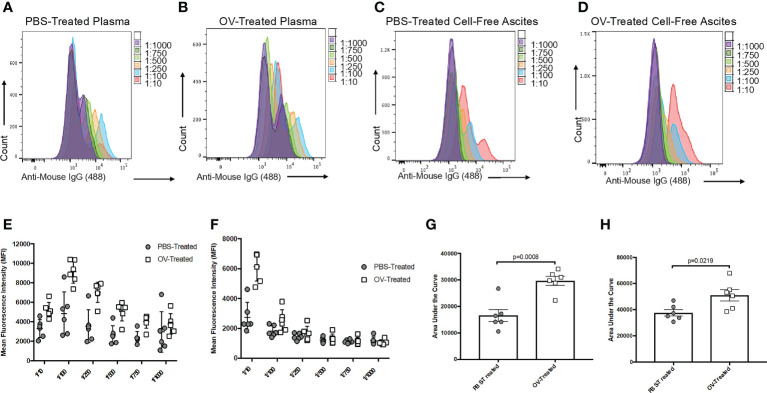
Optimization of the sample dilution scheme used to evaluate tumor-associated antibody responses following oncolytic virotherapy. Histogram overlays demonstrating the six-series dilution scheme of positive secondary antibody (anti-mouse IgG1 Alexa Fluor 488) signals from targeted ID8 cancer cells treated with serial dilutions of plasma from phosphate-buffered saline (PBS)-treated **(A)** or oncolytic virus (OV)-treated **(B)**, or serial dilutions of cell-free ascites fluid from PBS-treated **(C)** or OV-treated **(D)** ID8 tumor-bearing mice. Curves were generated from the mean fluorescence intensities (MFIs) of the Alexa Fluor 488 signals from targeted ID8 cells treated with serial dilutions of plasma **(E)** or cell-free ascites fluid **(F)** collected 21 days following treatment of ID8 tumor-bearing mice with PBS or OV. Each data symbol represents a diluted sample from a unique biological replicate (*n*=6 mice per group). Curves generated from the MFI for each biological replicate were used to calculate the area under the curve for comparison of tumor-associated antibodies from PBS-treated and OV-treated mice in the plasma **(G)** and cell-free ascites fluid **(H)**. Statistical analysis was conducted using a two-tailed Student’s t-test. Statistical significance was defined as P-values less than or equal to 0.05.

The sensitivity of this assay relies on optimization of the dilution of the samples and secondary detection antibodies. At extremely high sample concentrations, there is an increased risk of non-specific binding of antibodies within samples to target cells, creating a threshold in terms of how much fluorescence can be detected. To resolve this, it is recommended that a range of sample dilutions are included for each sample in each experiment, especially if relatively high-magnitude antibody responses are expected. **Figure 3** shows results using an ideal range for plasma and cell-free ascites fluid samples from PBS- and OV-treated tumor-bearing mice that facilitated relative quantification of antibodies by calculating areas under the curves, which is used to compare peaks in fluorescence outputs that correlate with antibodies in samples. To confirm the selection of sufficient dilutions, samples can be visualized by making use of the overlay feature in the FlowJo software, as depicted in **Figures 3A–D**. Ideally, non-targeted cells, target cells only, and target cells incubated with secondary detection antibodies should be distinguished by a relatively low fluorescence intensity, while positive test samples would have a relatively high fluorescence intensity.

Non-specific binding of secondary antibodies can also occur, especially when they are used at high concentrations. Each fluorochrome-conjugated secondary antibody of interest should be tested at a range of dilutions with target cells only to determine a dilution that fails to yield a significant fluorescent signal on the flow cytometer. This ensures optimal detection of antigen-specific antibodies in experimental samples that have bound to target cells.

##### Standardizing fluorescent output and quantification

To provide a more accurate depiction of the antibody response to an immunotherapy by utilizing this protocol, it is recommended that positive secondary antibody signals be quantified based on MFI. This is because MFI values indicate the density of fluorochrome-conjugated secondary antibodies bound to target molecules on a per cell basis (28, 29). Attempted quantification based on the percentage of positively stained cells would not be accurate.

In this study we used autologous tumor cells as natural sources of undefined tumor-associated antigens or permissive non-tumor cells infected with an antigen-expressing viral vector to induce expression of a pre-specified target antigen. To ensure accurate comparisons of antibody concentrations between different samples, it is important that the target cells in each well express similar quantities of antigens. As such, using a single batch of cells within a given assay is important. Whenever possible it is recommended that a large batch of target cells be stored frozen as single-use aliquots that get thawed, passaged once and then used as is or then infected with an antigen-expressing virus and then used. This will maximize the consistency of results between different experiments. When the antigenic target is known, it is recommended to confirm uniform expression of reasonable concentrations of the antigen in or on cells prior to using this assay in a project. This can be accomplished by western blotting and use of an immunofluorescence assay.

## Results

### Detecting tumor-associated antibody responses in tumor-bearing mice treated with an OV

To test this protocol for detecting tumor-associated antibodies, a C57BL/6 murine model of orthotopic, syngeneic ID8 epithelial ovarian carcinoma was used as previously described (30, 31). Sixty days following the implantation of ID8 tumor cells under the ovarian bursa, mice were injected intraperitoneally with an OV known as Orf virus (OrfV) (32). Twenty-one days following treatment, blood and ascites were sampled, and plasma and cell-free ascites fluid were harvested for attempted detection of antibodies. Autologous ID8 cells were used as targets for potential binding of tumor-associated antibodies, and samples were diluted following the dilution series shown in **Figures 3A–D**. Samples were run through this protocol, including the intra-assay controls that were previously described. Both plasma (**Figures 3E, G** **)** and cell-free ascites fluid (**Figures 3F, H** **)** samples collected from tumor-bearing control mice that were treated with PBS yielded lower detection-antibody-mediated fluorescent signals following removal of background fluorescence. This is indicative of low-magnitude, tumor-induced IgG1 antibody responses. In contrast, mice treated with the OV had evidence of higher magnitude tumor-associated IgG1 antibody responses, as evidenced through greater MFI signals and area under the curve values (**Figures 3E–H**). These results demonstrate the efficiency of this assay in detecting treatment-induced changes in the antibody repertoire following administration of an immunotherapy expected to induce a robust antibody response. It is important to note that these antibody responses were ‘therapy-induced’ or ‘tumor-associated’ and not necessarily tumor-specific, as some target antigens could potentially be shared with off-target normal cells expressing the same antigen(s).

### Differentiating OV-induced tumor-associated antibody responses by isotype

**Figure 4:**
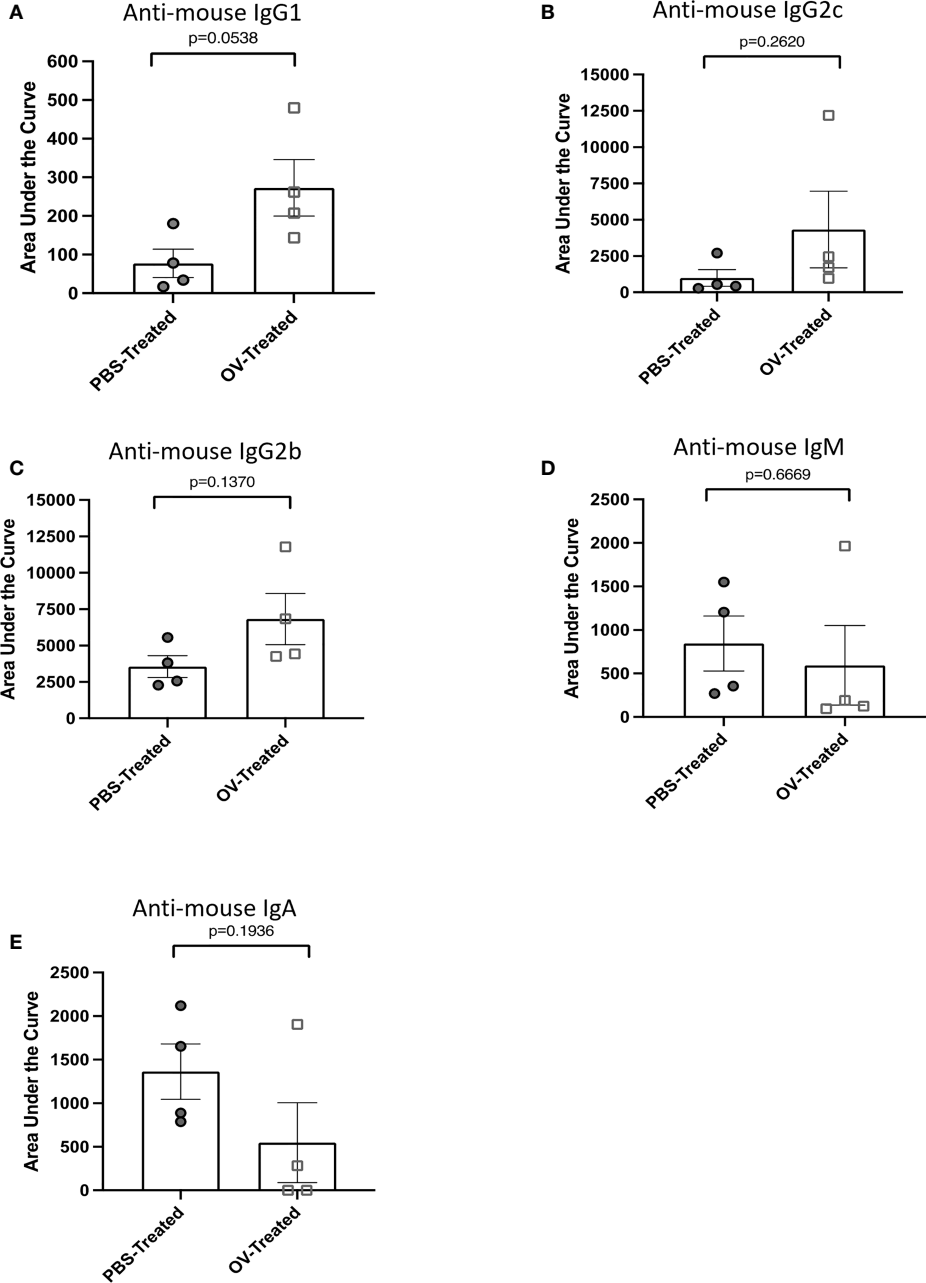
Multiplex assessment of tumor-associated antibody isotypes following oncolytic virotherapy. Tumor-associated antibody responses were quantified by flow cytometry on cell-free ascites fluid collected from ID8 tumor-bearing mice 21 days following treatment with phosphate-buffered saline (PBS) or an oncolytic virus (OV) (*n*=8 mice per group). Tumor-associated antibody binding was represented as a function of positive anti-mouse IgG1-APC, IgG2c-Alexa Fluor 488, IgG2b-PE-Cy7, IgM-PerCP-Cy5.5, or IgA-BV421-conjugated secondary antibody signals from target ID8 cells. Curves generated from the mean fluorescence intensity of each positive secondary antibody signal from targeted ID8 cells treated with four-series dilution scheme of cell-free ascites fluid samples were used to calculate the areas under the curves. The area under the curve was used to assess the tumor-associated antibody response of **(A)** IgG1, **(B)** IgG2c, **(C)** IgG2b, **(D)** IgM and **(E)** IgA isotypes within the cell-free ascites fluid of PBS- or OV-treated mice. Statistical analysis was conducted using a two-tailed Student’s t-test. Statistical significance was defined as P-values less than or equal to 0.05.

To demonstrate the potential of this assay in simultaneously detecting therapy-induced antibody responses of varied isotype in a multiplex assay, C57BL/6 mice bearing orthotopic ID8 ovarian cancers were treated with OrfV 60 days following tumor implantation. Ascites fluid was collected and harvested 21 days following treatment. Autologous ID8 cells were used as the target cells. Following dilution of samples, they were simultaneously analyzed for IgG1 (**Figure 4A**), IgG2c (**Figure 4B**), IgG2b (**Figure 4C**), IgM (**Figure 4D**), and IgA (**Figure 4E**) tumor-associated antibody responses by using distinct fluorochrome-conjugated secondary isotype-specific antibodies. While no statistically significant differences were observed for each individual antibody isotype within the peritoneal cavity between PBS- and OV-treated mice, OV-treated mice exhibited higher magnitude IgG1, IgG2c and IgG2b tumor-associated antibody responses (**Figures 4A–C**). Antibodies can have variable functional capacities, binding avidities and therapeutic effects dependent on the specific isotype, and it is largely speculated that having a broader repertoire of tumor-associated antibody isotypes may contribute to an increased quality in the overall anti-tumor immune response elicited (33, 34). As a result, the capacity of this protocol to detect multiple subclasses of immunoglobulin responses induced by a given immunotherapy platform is highly relevant for application in clinical settings for determining which immunoglobulin subtypes are contributing to therapeutic efficacy.

### Detecting virus-associated antibodies

**Figure 5:**
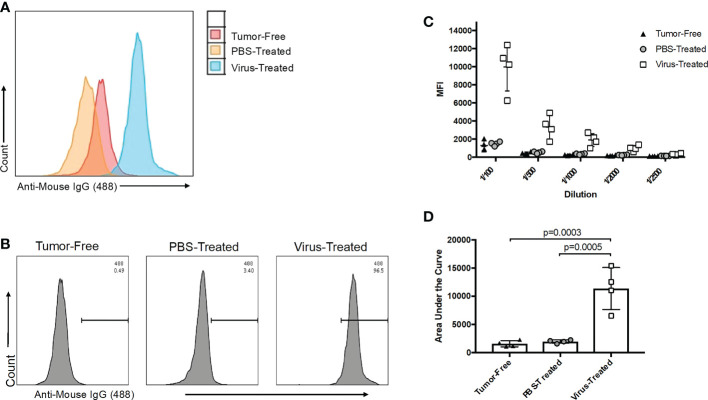
Evaluation of oncolytic virus (OV)-associated antibody responses. **(A)** Representative histogram overlay of target Vero cells infected for 16 hours with an OV and treated with a 1/100 dilution of plasma collected 21 days post-treatment from tumor-free (red), phosphate-buffered saline (PBS)-treated ID8 tumor-bearing (orange) or OV-treated ID8 tumor-bearing mice (blue) (*n*=4 mice per group). OV-associated antibody binding was represented as a function of positive anti-mouse IgG1 Alexa Fluor 488 conjugated secondary antibody signals from targeted OV-infected cells. **(B)** Representative histograms of OV-associated antibodies binding to targeted OV-infected Vero cells. The antibodies were derived from plasma samples diluted 1/100 after collection from tumor-free, PBS-treated ID8 tumor-bearing or OV-treated ID8 tumor-bearing mice 21 days post-treatment. The data shown are representative of positive anti-mouse IgG1 Alexa Fluor 488 conjugated secondary antibody signals from OV-infected target cells. **(C)** Data derived from the five-series dilution scheme (of which only one dilution was shown in panel B) were used to generate curves for each biological replicate. **(D)** Areas under the curves from data shown in panel C were calculated and used for comparison of virus-associated antibodies from the plasma of untreated tumor-free, PBS-treated, and OV-treated tumor-bearing mice. Statistical analysis was performed by one-way analysis of variance. Statistical significance was defined as P-values less than or equal to 0.05.

Due to the immunogenic nature of viruses, immunotherapy platforms designed with viral backbones, such as virus-vectored vaccines or OVs, can stimulate undesired virus-associated immune responses that neutralize and/or eliminate the virus, thereby limiting treatment efficacy upon re-administration. This can be a particular problem for OV-based platforms, as these often require multiple administrations to achieve optimal therapeutic effects. To decipher whether the method presented here could be used to discern virus-associated antibody responses, tumor-free C57BL/6 mice and orthotopic ID8 ovarian tumor-bearing mice were treated with PBS or OrfV 60 days following tumor implantation. Plasma was collected 21 days following treatment. Vero cells, which are permissive to OrfV infection, were used as targets, given that they share limited antigens with murine ID8 tumor cells and can readily express OrfV-derived antigens following short infection periods. Vero cells were infected with OrfV at a multiplicity of infection (MOI) of three for 16 hours to ensure that most cells would be exposed to the virus to maximize production of viral proteins but without a substantial amount of virus-induced cell death occurring. The method described in this paper was then conducted, with the inclusion of uninfected Vero cells as the off-target cell control. Plasma samples from tumor-free mice treated with OrfV were included in the assessment to control for the potential binding of tumor-specific antibodies present within samples to Vero antigens that may be shared with ID8 cells. Mice treated with OrfV had potent virus-associated IgG1 responses, as depicted by an overall increase in fluorescent values compared to plasma from untreated tumor-bearing or tumor-free mice (**Figure 5**). These results showcase the ability of this technique to detect virus-associated antibody responses against the entire repertoire of viral antigens, allowing for the acquisition of a global picture of the potentially limiting vector-specific antibody response. While antibody binding detected by this assay can contribute to clearing viruses *in vivo* by enhancing phagocytosis, opsonization, antibody-dependent cellular cytotoxicity or complement-mediated cytotoxicity of virus-infected cells, it is not always associated with virus neutralization activity. As such, we recommend combining this protocol with more traditional assays to identify viral neutralization and other functional capacities of antibodies.

### Detecting antigen-specific antibody responses in mice treated with an adenovirus-vectored vaccine against severe acute respiratory syndrome-coronavirus-2

**Figure 6:**
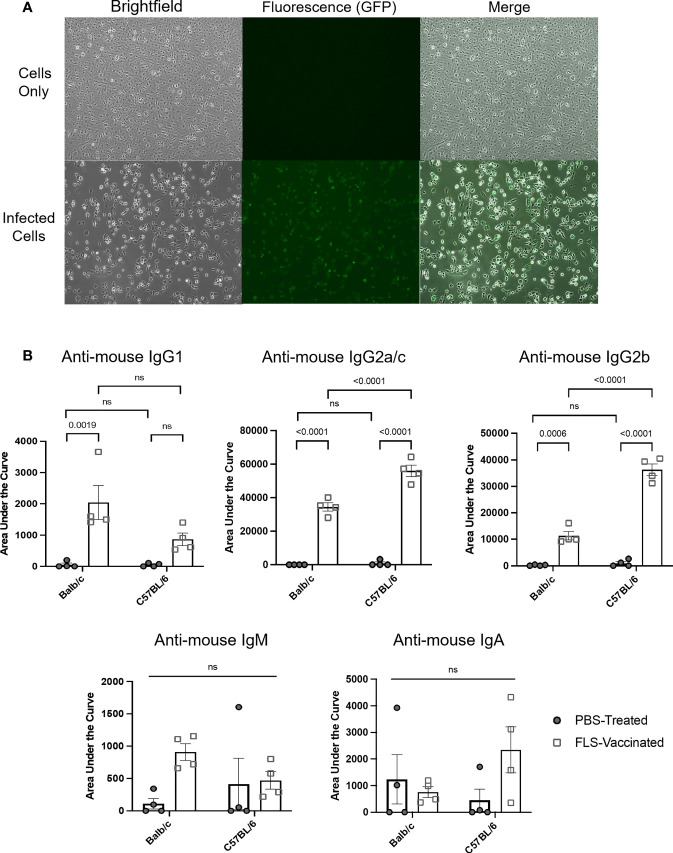
Detection of antibody responses induced by a viral-vectored vaccine platform targeting a pre-defined antigen. **(A)** DF-1 cells were treated for 16 hours with media only or infected at a multiplicity of infection of three with a recombinant avian orthoavulavirus-1 (AOaV-1) expressing the full-length spike protein (FLS) from severe acute respiratory syndrome-coronavirus-2. Spike protein expression on target cells was assessed using an immunofluorescence assay following incubation of cells with a murine primary AOaV-1 ribonucleoprotein-specific antibody diluted 1/2,000, and a secondary goat-anti-mouse conjugated with Alexa Fluor 488 diluted 1/1,000. Brightfield and fluorescent images were captured with an inverted fluorescent microscope at 20x magnification, with identical exposures for uninfected and infected cells. Target cells were expressing spike protein based on evidence of an Alexa Fluor 488 signal. **(B)** Disease-free Balb/c and C57BL/6 female mice were inoculated intramuscularly with phosphate-buffered saline (PBS) or a recombinant adenovirus expressing FLS (Ad-FLS). Plasma was collected 21 days post-vaccination and analyzed for isotype-specific antibodies directed against the spike protein using AOaV-1-FLS-infected DF-1 cells as the source of the target antigen. Spike protein-associated antibody binding was represented as a function of positive anti-mouse IgG1-APC, IgG2a/c-Alexa Fluor 488, IgG2b-PE-Cy7, IgM-PerCP-Cy5.5, or IgA-BV421-conjugated secondary antibody signals from target cells. Mean areas under the curves were calculated following determination of mean fluorescence intensities for all plasma dilutions tested. These were plotted and compared to evaluate antigen-specific, therapy-induced antibodies bound to antigen-expressing target cells. Statistical analysis was conducted by two-way analysis of variance. Statistical significance was defined as P-values less than or equal to 0.05. ns = "not significant".

To expand the use of this methodology for evaluating vaccine-induced antibody responses to a pre-defined antigen, female Balb/c and C57BL/6 mice were inoculated either intramuscularly with a recombinant adenovirus expressing the full-length human SARS-CoV-2 spike protein (Ad-FLS), or intranasally with Ad-FLS, or with PBS. To express the known target antigen, in this case the SARS-CoV-2 spike protein, in a cell line that normally lacks expression of the protein, DF-1 cells were infected at an MOI of three with a heterologous recombinant avian orthoavulavirus-1 (AOaV-1) expressing the full length SARS-CoV-2 spike protein [AOaV-1-FLS (35)] for 16 hours. Spike protein expression on target cells was assessed with an immunofluorescence assay as previously described (35), and target cells were discerned as positive for spike antigen expression based on Alexa Fluor 488 signaling (**Figure 6A**). Plasma was collected 21 days post-vaccination and analyzed utilizing the protocol described here for isotype-specific antibodies directed against the spike protein, with AOaV-1-FLS-infected DF-1 cells as the source of the target antigen. Plasma samples were simultaneously analyzed using uninfected DF-1 cells as a control to account for the potential binding of non-antigen-specific antibodies present within samples to DF-1 antigens. Following sample dilution, samples were analyzed for IgG1, IgG2a/c, IgG2b, IgM, and IgA responses simultaneously by use of distinct fluorochrome-conjugated secondary antibodies to evaluate antigen-specific, vaccine-induced antibodies bound to antigen-expressing target cells. Balb/c mice vaccinated with Ad-FLS had significantly higher concentrations of antigen-specific IgG1, IgG2a and IgG2b antibodies in comparison to unvaccinated mice, but not IgM or IgA (**Figure 6B**). Ad-FLS-vaccinated C57BL/6 mice displayed significantly greater amounts of antigen-specific IgG2c and IgG2b antibodies in comparison to unvaccinated mice, but not IgG1, IgM or IgA. Interestingly, vaccinated C57BL/6 mice exhibited significantly higher amounts of IgG2 responses compared to vaccinated Balb/c mice, suggestive of a T-helper cell-1-biased immune response in this strain.

The experiments presented herein demonstrate the utility of this method as a rapid and effective alternative to other techniques for assessing the broad spectrum of antibody responses induced by vaccination to pre-defined antigens on an isotype-specific basis. In doing so, this method can be used to analyze class-switching and type 1 versus type 2 immune response bias induced by an immunotherapy or vaccine. Both these immunological parameters contribute towards dictating therapeutic outcomes.

### Standardizing fluorescent output to quantify antibody responses

**Figure 7:**
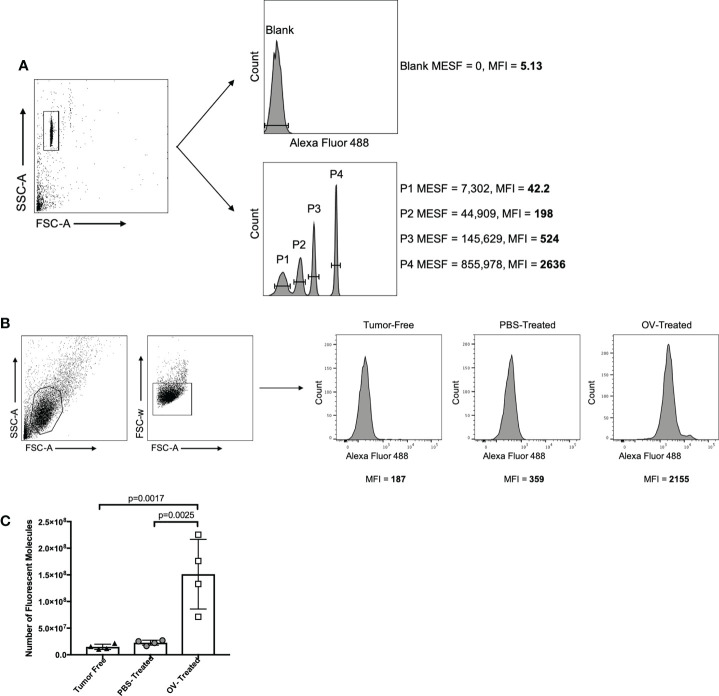
Strategy for standardizing fluorescent output using Quantum^TM^ molecules of equivalent soluble fluorochrome (MESF; Bang Laboratories). **(A)** Quantum^TM^ Alexa Fluor 488-conjugated beads were isolated based on forward scatter area (FSC-A) versus side scatter area (SSC-A) characteristics, and the mean fluorescence intensity (MFI) of each bead population containing a defined number of molecules of MESF was determined by flow cytometry. **(B)** In the same experiment, target oncolytic virus (OV)-infected Vero cells were identified by graphing FSC-A versus SSC-A, and doublets were excluded by graphing FSC-A versus forward scatter width (FSC-W). The MFI of the anti-mouse IgG1-Alexa Fluor 488 signal was then determined for target cells treated with a 1/250 dilution of plasma from tumor-free, phosphate-buffered saline (PBS)-treated tumor-bearing or OV-treated tumor-bearing mice. Virus-associated antibody binding was represented as histograms with cell counts on the y-axis and the intensity of the secondary Alexa Fluor 488-conjugated mouse IgG1-specific antibody signal on the x-axis. **(C)** The MFI of each Quantum^TM^ bead population was plotted against the defined MESF number to generate a standard curve. The MFI of anti-mouse IgG1 Alexa Fluor 488 from targeted OV-infected Vero cells treated with plasma from different treatment groups was plotted against the standard curve to estimate the number of anti-mouse IgG1-Alexa Fluor 488 molecules bound, which was then multiplied by the 1/250 dilution factor to approximate the number of virus-associated antibodies present in the original plasma sample.

To enhance the consistency and accuracy of quantification of immunotherapy-induced antibodies using the method described here, commercially available standardized beads were purchased. These beads had a pre-determined fluorescence intensity and were tagged with the same fluorochrome as the secondary detection antibody used to detect therapy-induced antibodies bound to target cells. Specifically, Bang Laboratories Quantum Alexa Fluor 488-conjugated beads were used. These beads included five different microsphere populations that were labeled with distinct concentrations of Alexa Fluor 488-conjugates, the use of which has previously been well characterized (36, 37). In brief, the fluorescence intensity of each bead population in the mix is correlated to the precise surface occupancy of Alexa Fluor 488 on each bead standard, referred to as the MESF value. Beads were prepared according to the manufacturer’s instructions and were run on the same day and at the same flow cytometer photomultiplier tube voltages and compensation settings as the mouse-derived samples being tested. This was to ensure the calibration curve used to extrapolate the number of fluorescent molecules could be used to accurately quantify antibody concentrations in test samples. The FSC-A and SSC-A settings on the flow cytometer were adjusted to clearly visualize the calibration beads. The voltage setting of the relevant photomultiplier tube was adjusted as needed so that fluorescent peaks representative of each microsphere population were clearly discernable (**Figure 7A**). A calibration curve was then generated using Bang Laboratories’ quantitative analysis software, QuickCal^®^, whereby the MESF value of each microsphere population was correlated to the MFI value observed. Within the same experiment, plasma collected 21 days following treatment of tumor-free and ID8 ovarian tumor-bearing C57BL/6 mice with PBS or OrfV was analyzed for evidence of OrfV-associated antibodies. Vero cells were infected with OrfV at an MOI of three for 16 hours and were incubated with serial dilutions of plasma samples following the protocol described in this paper. Gating of target cells on the flow cytometer was done based on FSC-A and SSC-A characteristics. Doublets were excluded by plotting FSC-A versus FSC-W. The anti-mouse IgG1 Alexa Fluor 488-derived fluorescent signal and respective MFI value corresponding to virus-associated antibody binding was then determined for target cells treated with each respective sample (**Figure 7B**). The MFI values from each individual test sample were graphed against the Quantum bead calibration curve, allowing the fluorescence intensity of each sample to be quantified as a distinct MESF value, representative of the number of bound Alexa Fluor 488 fluorescent molecules that would correlate with the number of virus-associated antibodies within each sample. As expected, mice treated with OrfV had the highest number of virus-associated IgG1 antibodies compared to PBS-treated tumor-bearing or tumor-free mice (**Figure 7C**). These results highlight how this assay can be used to accurately quantify and compare antibody responses across different experiments using standardized microsphere populations. Conceptually, this could be applied to quantifying fluorescent signals across multiple fluorophores, depending on the availability of relevant and distinct fluorochrome-conjugated bead standards. As such, this methodology can be practically applied to determine the absolute number of isotype-specific antibodies directed against any antigen of interest.

## Discussion

This protocol builds on our previously established methodology for detecting and quantifying tumor- and virus-associated antibodies following immunotherapy (27). This flow cytometry method can be used as an alternative to other techniques for assessing antibody responses following antigen-agnostic therapies such as OVs, whereby the use of adherent or suspended autologous tumor cells as targets allows for all relevant tumor antigens (excluding those generated *de novo*) to be represented. The method can also be used following antigen-specific therapies where the pre-defined target antigen is expressed in a cell line that normally lacks expression of the protein. In theory, the procedures provided herein can be used to assess the full breadth of antibody responses elicited by any given immunotherapy. It offers several additional advantages such as rapid high-throughput readout, cost-effectiveness, sensitivity, versatility due to compatibility with several sample sources, isotype-specific Ig detection, and multiplex capabilities. While conventional immunological techniques used to detect antibodies, such as the enzyme-linked immunosorbent assay, can equally be applied for quantification of antibody concentrations, these frequently lack the sensitivity necessary to detect target antigens expressed at low concentrations on cells, to detect antigens that are not pre-defined, or to detect low numbers of cells that express the target antigen. Additionally, these remain time- and sample-consuming, with typically only a single Ig-isotype against one antigenic target able to be measured at a time and at a higher cost. The method described here permits the simultaneous analysis of multiple antibody isotypes against either pre-defined or undefined bulk target antigens from a single, small-volume sample. With as little as 20 µL of input sample, any number of antibody isotypes and subtypes can be tested, depending on how many channels are available on a flow cytometer. In addition to the small volume of samples and reagents required, this assay has a relatively quick staining process and contains fewer overall steps than more traditional techniques. By using automation and a flow cytometer capable of high-throughput data acquisition, the entire repertoire of the antibody response to a given immunotherapy can be rapidly detected from 96 samples in a single experiment, within two hours.

Through the detection of multiple subclasses of Ig responses, this method can also be used to analyze class-switching and immune response biases induced by immunotherapies. Identification of type 1 vs type 2 immune response biases is relevant in the context of cancers, given that pre-malignant and malignant tissues have commonly been associated with suppressed T-helper cell-1 responses and enhanced T-helper cell-2 responses that correlate with tumor progression and subdued anti-tumor responses (38, 39). This bias is also an important consideration in vaccine development against intracellular pathogens such as viruses, given that the most effective responses against intracellular organisms are type 1 in nature (40, 41). As such, vaccines formulated to tilt the balance in favor of type 1 immunity are vital for eliciting more effective and rapid responses upon virus re-infection, particularly in scenarios of incomplete T cell-mediated protection.

The protocol presented here represents a valuable methodology that can be added to the toolbox of researchers to evaluate the role of endogenous antibodies induced by any given immunotherapy. The integration of this assay into routine pre-clinical and clinical assessments can support early detection of malignant cells or viral infections, establishment of prognoses for patients, assessment of treatment efficacy, and help inform the design and optimization of next-generation immunotherapies.

## Data availability statement

The raw data supporting the conclusions of this article will be made available by the authors, without undue reservation.

## Ethics statement

The animal study was reviewed and approved by Animal Care Committee, University of Guelph.

## Author contributions

Conception and design: JAM, JPvV, GAW, AMV-P, KK, JJP, SKW and BWB; development of methodology: JAM, JPvV, SKW and BWB; acquisition of data: JAM, JPvV, JGEY, LC, JJP; analysis and interpretation of data: JAM, JPvV, SKW and BWB; writing, review and/or revision of the manuscript: JAM, JPvV, GAW, AMV-P, KK, JJP, SKW and BWB All authors contributed to the article and approved the submitted version.

## Funding

Funding was provided by Discovery Grants from the Natural Sciences and EngineeringResearch Council of Canada to BB. (#436264) and SW (#499834), anOperating Grant from the Cancer Research Society to BB and SW(#843296), and an Operating Grant from the Pet Trust Foundation to KK(#054725). Stipend funding was from: Ontario Graduate Scholarship andNora Cebotarev & Ellen Nilsen Memorial Scholarship (for JM); CanadianGraduate Scholarship-Doctoral Award (NSERC), Ontario GraduateScholarship and Ontario Veterinary College (OVC) Graduate Scholarship (forJV); OVC Graduate Scholarship (for JY and LC).

## Acknowledgments

We thank Campus Animal Facilities, University of Guelph, for animal care services.

## Conflict of interest

B.W.B is the Chief Scientific Officer of ImmunoCeutica Inc., which is dedicated to the research and development of immunoceuticals. S.K.W. is a founder of Avamab Pharma Inc., which produces gene therapy vectors that express antibodies, and the senior scientific advisor for Cellastra Inc., which is dedicated to research and development of gene therapies targeting root causes of scarring. ImmunoCeutica Inc., Avamab Pharma Inc., and Cellastra Inc. did not provide any funding, nor did they have any role in the design of the study; in the collection, analyses, or interpretation of data; in the writing of the manuscript, or in the decision to publish the results.

The remaining authors declare that the research was conducted in the absence of any commercial or financial relationships that could be constructed as a potential conflict of interest.

## Publisher’s note

All claims expressed in this article are solely those of the authors and do not necessarily represent those of their affiliated organizations, or those of the publisher, the editors and the reviewers. Any product that may be evaluated in this article, or claim that may be made by its manufacturer, is not guaranteed or endorsed by the publisher.
