# Supplementary figures and images for: Multiplex flow cytometry-based assay for quantifying tumor- and virus-associated antibodies induced by immunotherapies
Source: Front Immunol. 2022 Nov 16;13:1038340. doi: 10.3389/fimmu.2022.1038340 (PMC9708883; doi:10.3389/fimmu.2022.1038340)

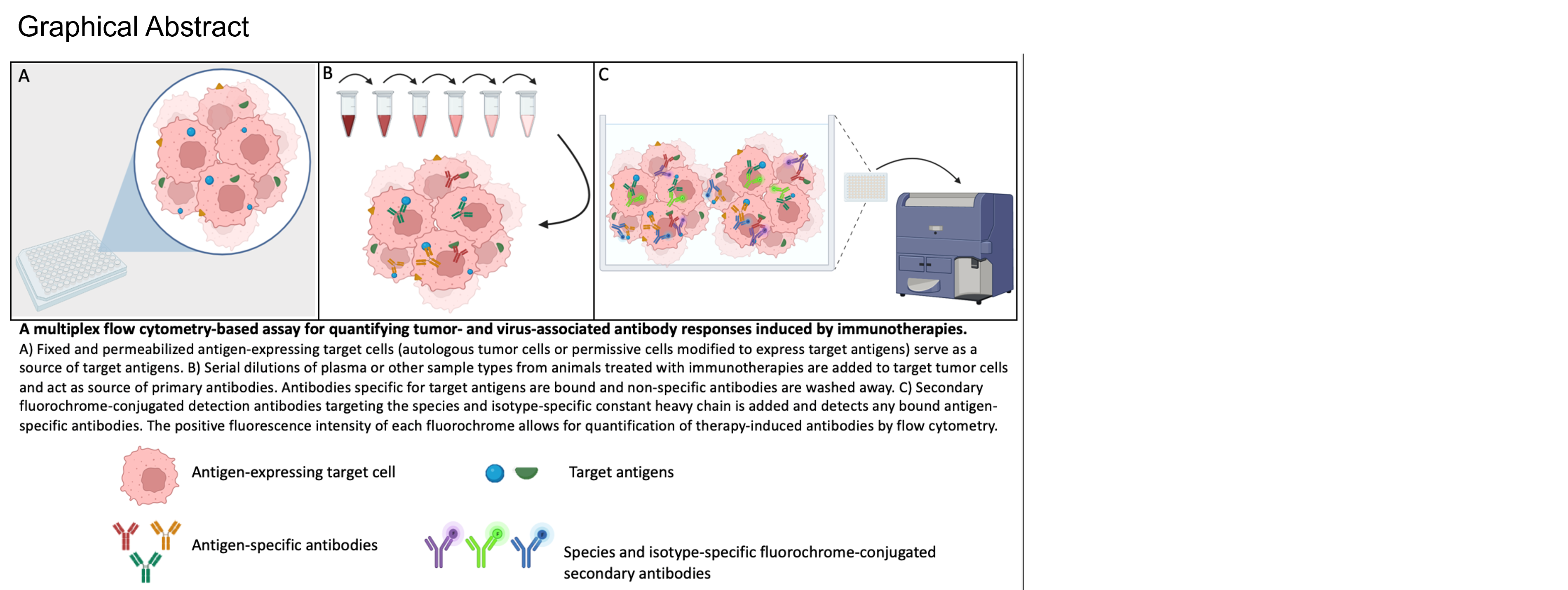

Supplement: Supplementary file 1 [file Image_1.tif]
