# Supplementary material for: Multiplex flow cytometry-based assay for quantifying tumor- and virus-associated antibodies induced by immunotherapies
Source: Front Immunol. 2022 Nov 16;13:1038340. doi: 10.3389/fimmu.2022.1038340 (PMC9708883; doi:10.3389/fimmu.2022.1038340)
Supplement: Supplementary file 2 [file Table_1.docx]

Manuscript ID Frontiers in Immunology-1038340

Response to Reviewer 1 Major concern 2

Addition of a Supplementary Table

| **Antibody Response Under Investigation** | **Sample Type** | **Target Cell** | **Analyses** |
| --- | --- | --- | --- |
| Antibodies against an oncolytic Orf virus that was used to treat orthotopic epithelial ovarian carcinomas in a pre-clinical murine model. | Heat-inactivated plasma from tumor-free, or ID8 epithelial ovarian carcinoma-bearing C57BL/6 female mice 21 days following treatment with oncolytic Orf virus or administration of phosphate-buffered saline. | Vero cells infected for 16 hours with Orf virus at a multiplicity of infection of three prior to fixation and permeabilization of cells. | Target cells were incubated with serially diluted plasma samples. Flow cytometry was used to determine fluorescence intensities derived from fluorochrome-conjugated anti-mouse IgG1 secondary antibodies bound to primary plasma-derived antibodies that had bound to viral antigens. The areas under the curves of graphs on which fluorescence intensities were plotted against plasma dilutions was used to quantify the antibody response. |
| Severe acute respiratory syndrome-coronavirus-2 (SARS-CoV-2)-derived spike protein-specific antibodies induced by a recombinant viral-vectored vaccine platform expressing the spike protein. | Heat-inactivated plasma collected from Balb/c and C57BL/6 female mice 21 days after inoculation with phosphate-buffered saline or a recombinant avian orthoavulavirus-1 (AOaV-1) expressing the full-length spike protein from SARS-CoV-2. | DF-1 immortalized chicken embryonic fibroblasts transfected with the spike protein from SARS-CoV-2 prior to fixation and permeabilization of cells. | Target cells were incubated with serially diluted plasma samples. Flow cytometry was used to determine fluorescence intensities derived from fluorochrome-conjugated anti-mouse IgG1, IgG2a/c, IgG2b, IgM, or IgA secondary antibodies bound to primary plasma-derived antibodies that had bound to spike proteins. The areas under the curves of graphs on which fluorescence intensities were plotted against plasma dilutions was used to quantify the antibody response. |
| ID8 epithelial ovarian carcinoma-associated antibodies induced by treatment with oncolytic Orf virus. | Heat-inactivated plasma and cell-free ascites collected from ID8 tumor-bearing C57BL/6 female mice 21 days following treatment with oncolytic Orf virus or administration of phosphate-buffered saline. | Fixed and permeabilized ID8 tumor cells. | Target cells were incubated with serially diluted plasma or ascites samples. Flow cytometry was used to determine fluorescence intensities derived from fluorochrome-conjugated anti-mouse IgG1, IgG2a/c, IgG2b, IgM, or IgA secondary antibodies bound to primary plasma-derived antibodies that had bound to antigens in and on the ID8 cells. The areas under the curves of graphs on which fluorescence intensities were plotted against plasma dilutions was used to quantify the antibody response. |

**Supplementary Table S1: Summary of experiments used to assess the potential of the flow cytometry-based assay to quantify antibody responses.**
